# Household Behavior with Respect to Meat Consumption: Differences between Households with and without Children

**DOI:** 10.3390/vetsci4040053

**Published:** 2017-10-31

**Authors:** Merlino Valentina Maria, Borra Danielle, Verduna Tibor, Massaglia Stefano

**Affiliations:** Department of Agricultural, Forest and Food Sciences, University of Torino, Largo Paolo Braccini 2, 10095 Grugliasco (TO), Italy; danielle.borra@unito.it (B.D.); tibor.verduna@unito.it (V.T.); stefano.massaglia@unito.it (M.S.)

**Keywords:** consumer behavior, household composition, meat attributes, Best-Worst analysis

## Abstract

Meat consumers around the world are increasingly paying attention to product quality and safety, and are starting to reduce their meat consumption, especially with regard to red meat. This trend is prevalent in households with children who prefer health-certified meat products. Our study compares meat consumption habits in households with and without children or adolescences (0–18 years). A structured questionnaire was distributed to 401 retail purchasers at 12 different points of sales of meat in the Piedmont region in northwest Italy. Socio-demographic variables and quantitative-qualitative meat consumption habits of retail purchasers were investigated. One part of the questionnaire analyzed the relative importance of 12 meat choice purchasing attributes by employing the Best-Worst scaling methodology, a type of choice experiment. Our research found that households without children (subset B) have higher weekly meat consumption habits than those with children (subset A). Alternatively, the households with children (subset A) have a diet characterized by a greater variety of protein sources, such as legumes and fish. Both of the considered subsets preferred trusted butchers for meat buying, with supermarkets as a second choice. However, only consumers of subset A bought meat from farm butchers. Our team performed a consumer analysis to identify meat consumption patterns in the two considered subsets. Simultaneously, a Best-Worst analysis evidenced several choice attributes with different relevance for the two investigated samples segmentation in three clusters of purchase.

## 1. Introduction

Global meat consumption is undergoing both a quantitative (negative trend) and qualitative change (shift towards white meat consumption) [1,2]. Overall, there has been a global reduction of red meat consumption, which has been partially replaced by leaner white meat. The analysis of consumer perception of meat attributes is important for understanding and predicting its behavior [3]; currently, consumer choice and purchasing decisions are oriented toward food safety, healthy products and quality characteristics [4,5,6]. However, meat experience-consumption characteristics and credence quality attributes influenced these processes, as well [7,8]. Moreover, consumer attitudes are influenced by values and social rules, which are determined by multiple aspects of everyday life for individuals or groups of people [9,10]. This aspect is confirmed in households with children, having a positive effect on quality certified meat consumption [1]. Meat consumption has been linked to the importance of a child’s growth. Research demonstrates that meat intake, both red and white meat, is positively associated with psychomotor development at 22 months of age [11]. In contrast, many studies have concentrated on the negative correlation between meat consumption at a young age and some chronic diseases, including obesity and cardiovascular disease, as adults. In fact, obesity is becoming a serious threat to both the immediate and long-term health of children. The effects of impaired nutritional status during childhood may have long-standing consequences for the health and performance of children during their adult years [12,13]. Therefore, the amount of meat intake recommended by pediatricians is important in maximizing benefits and limiting health risks [14,15]. It has become more apparent that consumers, often influenced by vague or biased media information, have changed their consumption of meat, especially in the presence of children [16,17].

The aim of this research was to evaluate whether or not meat consumption habits are influenced by household composition. A comparison of meat consumption habits was made between households with and without children or teenagers (0–18 years).

## 2. Materials and Methods

### 2.1. Data Collection

The study was conducted in the Piedmont region in northwest Italy. A structured questionnaire (consisting of closed-ended questions) was distributed to 401 retail purchasers at 16 meat points of sale (8 familiar points of sale of fresh-cut meat or trusted butchers; 6 meat points of sale of two mass retail channels where packaged, fresh and processed meat were sold; and 2 stores on breeding farms or farm butchers). The retailers were chosen both in rural and in metropolitan areas. The choice of different typologies of points of meat purchase was made in order to involve different targets of beef meat consumers. In addition to trusted butchers and mass retail channels, two farm butchers, which are more and more common in the considered geographic area, were also involved in the research.

Face-to-face interviews were conducted by using paper questionnaires (see Appendix A), alternating, whenever possible, between genders. The four different versions of the questionnaire were submitted randomly to the interviewees. Interviews were conducted every day from April to July 2015, from Monday to Sunday, in two time slots (9 a.m. to 1 p.m. and 4 p.m. to 8 p.m.).

### 2.2. Questionnaire Set-Up

The questionnaire was subdivided into three main sections. The first section included questions related to socio-demographic characteristics, such as age (under 30, from 31 to 45, from 46 to 55, and over 55), gender (female or male), educational status (primary school, lower secondary, upper secondary, master’s degree) and employment status (employed, retired, entrepreneur, student, unemployed or homemaker). The second section of the questionnaire was related to meat purchasing behavior and consumption. Quantitative and qualitative consumption patterns of meat, and beef meat in particular, were examined. The weekly consumption of meat and beef (no consumption, 1–2 times, 3–5 times, 6–10 times, and more than 10 times), as well as the habitual meat point of sale, and frequency (never, sometimes and often) of consumption of alternative protein sources (e.g., legumes and fish) were examined in this section. The third and last section analyzed the relative importance of 12 meat choice purchasing attributes (Table 1), which were selected after an in-depth review of articles published in international journals, by employing a choice experiment (the Best-Worst scaling methodology).

### 2.3. Best-Worst Methodology

The Best-Worst methodology was introduced by Finn and Louviere [18] in the early 1990s. It wasn’t until 2005 that a more rigorous methodological explanation was presented [19]. The Best-Worst (BW) methodology is a measuring technique in which respondents are asked to choose their favorite attribute (the best) and their least favorite attribute (the worst) from a set of attributes [20,21].

The BW score can be considered an extension of the pairwise comparison method, since it offers similar benefits and more information with fewer questions [22]. The BW methodology also provides a more discriminating way of measuring the degree of importance that respondents attach to each factor. For example, unlike the Likert scale, respondents can choose only two attributes which they consider as respectively the most and least important for each set of choice.

In accordance with [23], we chose to include 4 attributes per subset, and to present each attribute 3 times within the questionnaire (Table 2).

MaxDiff designer (v.2.0.2; Sawtooth Software, Orem, UT, USA) was used to assign each of the 12 attributes to 4 different versions of the questionnaire. Each of these versions comprised nine subsets, each including four attributes (see Appendix A).

A two-way balance was favored in this study design, meaning that it was directed towards the frequency with which paired combinations of attributes appeared together. As the average BW scores take positive and negative values, and therefore sum to zero, they are often perceived as difficult to interpret. For instance, in the case of importance measurement, a negative BW value does not indicate negative importance, but rather low (below average) importance [20]. Rescaling can be applied for the total sample or the subsamples, such as segments, to allow an easier comparison than using raw BW scores [20]. The total responses for each best and worst attribute were calculated using Sawtooth MaxDiff (SSI-version 8.4.6; Sawtooth Software, Orem, UT, USA, www.sawtoothsoftware.com) using the cyclical algorithm, k(k − 1)/2 (where k is the total number of attributes) making paired comparisons possible.

Furthermore, the Best-Worst analysis was used to understand if clusters with homogeneous preferences within the sample could be identified according to the weight that the individual respondent assigned to the different attributes, as per the Latent Class Clustering technique. The Sawtooth MaxDiff software, by default, creates 4 segmentations, each containing the division of the sample from 2 to 5 clusters respectively. For segmentation into clusters, the *p*-value for each attribute was calculated using the homogeneity of the variance test. The software used for the quantitative analysis was SPSS.21.0 for Windows (IBM Corporation, Armonk, NY, USA).

A data analysis was conducted comparing information provided from two subsets of the sample: consumers with children/teenager (0–18 years), designated as subset A; and families without children or teenager, designated as subset B.

Preferences of meat attributes by the considered consumer samples were analyzed by gender, age, educational level, employment, average weekly meat consumption, and the point of meat purchase.

## 3. Results

Socio-demographic characteristics of the two subsets, A and B, are reported in Table 3.

**Figure 1:**
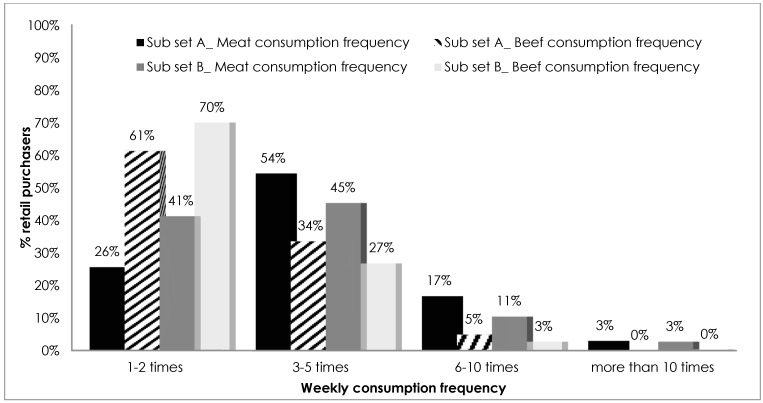
Weekly meat and beef consumption frequency in the considered subsets (A and B).

Out of the 401 respondents in the whole sample, 26% had at least one child or adolescent. In both subsets, there was a majority of women, especially in subset A (68% women versus 32% men). With regard to age, there were differences between the two considered groups. In subset A, the majority of respondents (66%) were between the ages of 31 and 45, only 2% were younger than 30 years, while in subset B, 33% were under 30, and 36% were between 31 and 45 years. In both groups, there were few respondents older than 55 years (3% and 8%, respectively, in subsets A and B). 63% of respondents from subset A had upper-secondary education, while 44% of subset B had a master’s degree. Differences in employment status was evident; 60% of subset A were considered entrepreneurs, while in subset B respondents were divided by being either employed (40%) or retired (31%). The majority of retail purchasers surveyed followed traditional meat-eating habits in both of the considered subsets (more than 95%). From the analysis of the responses, differences in consumption frequency emerged between the two considered subsets A and B (Figure 1), 54% of households with children consumed meat at least 3 times a week, and 45% of households without children consumed the same amount. One-fifth of sample A can be classified as high meat consumers (more than 6 times a week). Moreover, higher consumption of meat was associated with lower consumption of beef, and was compensated for by other types of meat. In fact, those who ate more meat per week, in proportion, ate less beef meat than those who ate meat 1 or 2 times weekly, which was 60–70% of beef.

**Figure 2:**
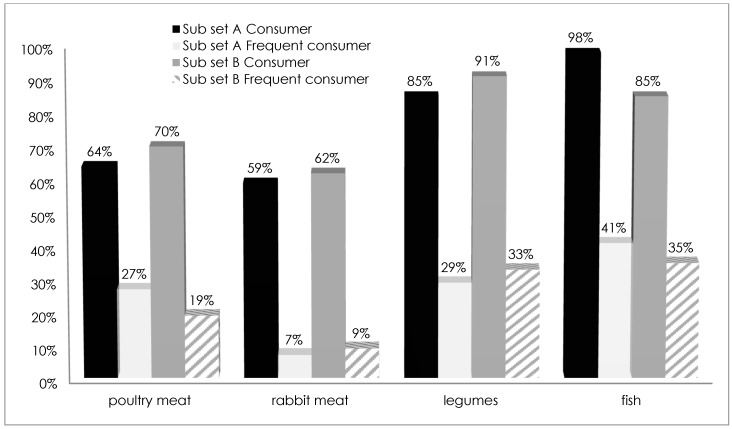
Protein sources: consumers and frequent consumers in subsets A and B.

**Figure 3:**
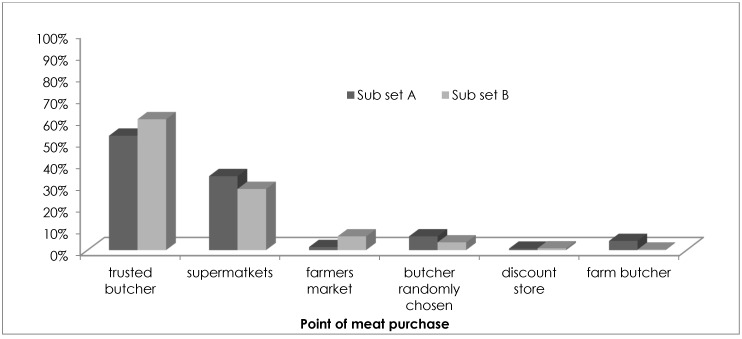
Different meat points of purchase chosen by consumers of subsets A and B.

The analysis of protein intake evidences some similarities in dietary styles of consumers in both of the subsets for poultry, rabbit and legumes. In contrast, fish was consumed more by subset A (Figure 2). Among the respondents, Figure 2 also shows the percentage of those who declared a “frequent” consumption of the various meat/protein sources proposed in the interview. The share of frequent consumers in sample A was higher than in sample B for poultry and fish. Regarding consumer preferences for beef meat cuts, no significant differences emerged from the analysis. The majority of retail purchasers in both of the considered subsets (73% and 72% in subsets A and B, respectively) bought beef meat from small meat butchers, followed by supermarkets. Only 1% of both subsets chose discount stores for meat purchase, while only consumers of subset A (6%) bought meat at farm butchers (Figure 3).

### Best-Worst Analysis

**Figure 4:**
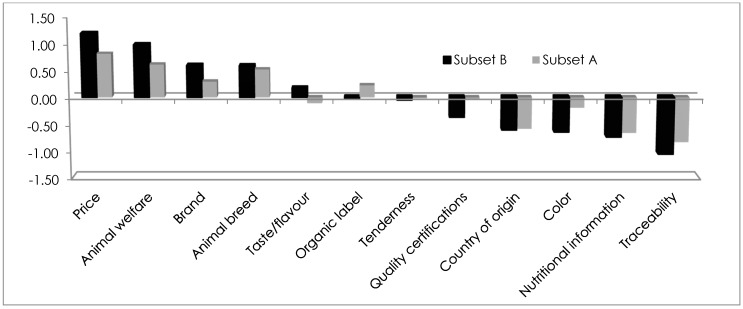
Best-Worst Analysis: preferences of beef meat attributes by the two considered subsets.

In Figure 4, meat attributes that are considered important during beef meat purchase are reported for both considered subsets. Price and animal welfare emerged as the most important attributes. Brand and animal breed, while not as significant, still have great importance for both of the samples. On the contrary, traceability, nutritional information, color and country of origin were the four least important choice attributes for subsets A and B. Different levels of importance are recognized in taste/flavor (more important for households without children) and organic labelling (important for households with children). Each subset was subdivided into clusters with both the data analysis to the Best-Worst method (Table 4).

In both cases, there is a “price sensitive” cluster, underscoring that consumers pay significant attention to the price when purchasing meat. Secondly, consumers place animal welfare as a significant attribute of choice, while the least relevant attribute is nutritional information. From the analysis of the importance of the different attributes of meat choice, traceability was revealed to be considered to be unimportant in clusters in both subsets A and B (households with and without children). Families with children view the brand as the most important attribute for cluster A1, while it is of medium importance in the clusters emerging in the subset of households without children. In A3, color is the most important attribute when choosing meat. The opposite situation is seen in cluster A1, where color is the least relevant attribute. Similarly, in families without children, we find in cluster B2 that color is not relevant, it is the taste/flavor that is the most important. In contrast, cluster B4 finds the color to be most important during the purchase of the meat, and considers the nutritional information the least relevant.

## 4. Discussion

A consumer analysis permitted us to identify meat consumption patterns and to quantify the importance of selected quality attributes at the purchasing stage in households with and without children. The sample involved in this study was small (401 interviewed); however, the choice experiment employed for investigation, the Best-Worst methodology, is feasible even for low size samples [24]. Households with children were likely to be concerned about nutritional balance in the diet [17]. Our research found that the presence of children in families affects the amount of meat consumed, especially beef, as well as the variety of diet, leaving space for other protein sources, such as legumes and fish. This result is in line with pediatricians’ recommended indications about the variety of diets and protein sources. Consumers with children are considered more likely to seek fortification in their foods [25]. The presence of children in families can influence the choice of food due to a potential association with a higher risk-aversion in food or a higher quality consciousness [26]. For example, a study conducted in the United States found that households with children consumed poultry and seafood less frequently in comparison with red meat. The same study noted that food safety concerns regarding seafood seemed to prevent parents from making seafood available for their children in their diets [17]. This was also apparent in Europe with regard to fresh meat after the BSE crisis, which caused a drastic reduction in consumption [27,28].

In our choice experiment, no significant difference in consumer behavior emerged at the point of meat purchase. Quality of product and safety guarantees were the two choices of equal importance at the point of meat purchase in both groups. In particular, as reported in other research, consumers assumed that all food in supermarkets has a safety guarantee [3,29]. Almost half of the households with children choose supermarkets to buy meat, and this is in agreement with the sample characteristics, especially with women and people who are employed. However, the trusted butchers were most frequently chosen to buy product that had been certified and guaranteed by the production specification “Piedmont beef”. The Best-Worst analysis showed that some choice attributes have different relevance for the two investigated samples. Seven of the twelve factors considered related to product description (price, animal welfare, animal breed, brand, organic label, taste/flavor and tenderness) were selected by the respondents as most important. The remaining factors were considered less relevant by consumers during meat purchase. Among the above-mentioned meat attributes, price and animal welfare emerged as the most important attributes. In this regard, note how the clusters’ segmentation of the sample has been identified as the “Price-sensitive” group, the most-represented group by respondents in both subsets A and B. Already in [8], consumers considered the price as an “extremely important” attribute during meat choice. Also, [30,31] confirm our results, which justify identifying the price consideration as being above all other meat characteristics, such as origin or nutritional aspects. However, this latter result is in contrast with the declarations reported in other studies, in which price attribute is less important than origin, as well as information regarding animal treatment and organoleptic aspects [32,33]. Brand and animal breed have great importance for both the samples. On the other hand, attributes such as traceability, nutritional information, color and country of origin are the least important for subsets A and B. Levels of importance vary from households with and without children. For example, taste/flavor is more important for households without children and organic labelling is important for households with children. In our research, the high quality of the considered product (in many cases of Piedmontese cattle breed) reduces the importance of the evaluation of some aspects (organoleptic quality, certifications, origin) that are generally considered important for European families with children [4], making the meat price the discriminating attribute during purchase. The current economic crisis has influenced consumers to focus their attention on the price of products and on the quality–price correlation. Furthermore, recent studies in the EU indicate that consumers are willing to eat animal-friendly food because they associate it with higher quality and health [34,35].

## 5. Conclusions

The present study investigated household behavior with respect to meat consumption, and evidenced several differences between the two investigated subsets: households with (subset A) and without children (subset B). Households without children have higher weekly meat consumption habits than ones with children. Alternatively, the households with children (subset A) have a diet characterized by a greater variety of protein sources. Our research found that trusted butchers represented the most used and preferred sales channel for both the analyzed subsets. The consumer analysis performed with a choice experiment permitted to identify meat consumption patterns and to quantify the importance of selected quality attributes at the purchasing stage. The results of Best-Worst Analysis evidenced that some choice attributes have different relevance for households with and without children. Overall, price and animal welfare are the most important attributes for meat choice for all the consumers involved in the study. Brand and animal breed have great importance for both of the samples. On the other hand, attributes such as traceability, nutritional information, color and country of origin are least important for subsets A and B. Relevant differences can be noticed for two beef meat attributes: taste/flavor is more important for households without children and organic labelling is important for households with children. The clusters’ segmentation identified the “Price sensitive” cluster in both of the two subsamples as the most represented.

## Figures and Tables

**Table 1:** Meat quality attributes used for the Best-Worst analysis.

Meat Qualitative Attributes		
Price	Brand	Animal welfare
Country of origin	Color	Taste/flavor
Traceability	Nutritional information	Tenderness
Animal breed	Organic label	Quality certifications

**Table 2:** Example of attributes subset. Respondents had to indicate which of the four presented attributes was considered the best and which worst.

Most Influential	Attributes	Least Influential
⚪	Tenderness	⚪
⚪	Certifications	⚪
⚪	Brand	⚪
⚪	Animal welfare	⚪

**Table 3:** Socio-demographic characteristics of the subsets A and B.

		Subset A	Subset B
**Gender**	Women	68%	58%
	Men	32%	42%
**Age**	≤30 years old	2%	33%
	Between 31 and 45 years old	66%	36%
	Between 46 and 55 years old	29%	23%
	>55 years old	3%	8%
**Education Level**	Primary School	0%	10%
	Lower Secondary School	10%	37%
	Upper Secondary School	63%	9%
	Master’s degree	27%	44%
**Employment Status**	Homemaker	20%	9%
	Unemployed	6%	2%
	Employed	12%	40%
	Entrepreneur	60%	14%
	Retired	0%	31%
	Student	2%	5%

**Table 4:** Importance of attributes for each cluster in the two subsets: Households with children (A) and households without children (B) *.

	Households with Children (A)			Households without Children (B)			
Attributes	Cluster A1	Cluster A2	Cluster A3	Cluster B1	Cluster B2	Cluster B3	Cluster B4
	30.1%	44.5%	25.5%	23.4%	32.5%	31.2%	12.9%
Traceability	7.59	2.48	2.80	3.60	7.66	2.05	8.03
Price	7.19	19.87	11.68	9.35	9.75	19.52	6.17
Brand	12.04	10.21	9.93	9.86	7.89	11.00	7.00
Animal breed	8.54	10.74	12.36	13.27	6.99	13.40	9.18
Color	4.40	2.87	12.78	11.05	3.76	4.79	18.00
Animal welfare	11.77	15.09	10.45	7.08	12.88	14.03	7.02
Country of origin	10.73	4.81	1.99	3.46	7.16	4.26	7.58
Organic label	4.49	10.12	7.74	7.84	7.65	10.36	11.10
Nutritional information	6.60	1.84	12.43	16.87	4.90	2.02	2.65
Tenderness	8.26	9.50	4.04	4.41	10.54	8.40	6.38
Quality certifications	8.01	4.41	6.87	8.43	7.73	5.13	11.75
Taste/flavour	10.38	8.08	6.93	4.78	13.08	5.04	5.13

* Importance of attributes: 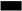 Most relevant attribute; 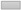 Second most relevant attribute; 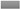 Least relevant attribute.

## Appendix A. Questionnaire

First Section: Respondent’s socio-demographic profile.

1. Gender □ Man □ Woman. Age: ________

2. Educational status:

□ Primary school	□ Lower secondary school	□ Upper secondary school	□ Bachelor or Master’s Degree

3. Employment

□ Student	□ Employed	□ Entrepreneur
□ Retired	□ Unemployed	□ Homemaker

Second Section: Respondent’s meat purchasing behavior.

4. Meat weekly consumption

□ No consumption	□ 1–2 times	□ 3–5 times	□ 6–10 times	□ More than 10 times

5. Beef meat consumption

□ No consumption	□ 1–2 times	□ 3–5 times	□ 6–10 times	□ More than 10 times

6. Different protein sources consumption frequency

• Poultry meat	□ never	□ sometimes	□ often
• Rabbit meat	□ never	□ sometimes	□ often
• Legumes	□ never	□ sometimes	□ often
• Fish	□ never	□ sometimes	□ often

Third Section: BEST WORST SCALING subset (Abbreviated example of one of the set of meat attributes submitted to the consumer.)

Please try to focus on your last beef meat purchase.

For each of the following tables, select the ONE reason that MOST influenced your choice and the ONE that LEAST influenced your choice.

**MOST/BEST (Only One Answer)**	**Attribute**	**LEAST/WORST (Only One Answer)**
⚪	Traceability	⚪
⚪	Price	⚪
⚪	Brand	⚪
⚪	Animal breed	⚪
