# Household Behavior with Respect to Meat Consumption: Differences between Households with and without Children. *Vet. Sci.* 2017, *4*, 53

**DOI:** 10.3390/vetsci6010012

**Published:** 2019-01-22

**Authors:** Valentina Maria Merlino, Danielle Borra, Tibor Verduna, Stefano Massaglia

**Affiliations:** Department of Agricultural, Forest and Food Sciences, University of Torino, Largo Paolo Braccini 2, 10095 Grugliasco (TO), Italy; danielle.borra@unito.it (D.B.); tibor.verduna@unito.it (T.V.); stefano.massaglia@unito.it (S.M.)

Due to an error during production, the order of the first and last names of the authors are incorrect in the published paper [1]. The correct names are as follows: Valentina Maria Merlino, Danielle Borra, Tibor Verduna and Stefano Massaglia. 

These changes do not affect the scientific results. The manuscript will be updated and the original version will remain online on the article webpage, with a reference to this erratum. The authors would like to apologize for any inconvenience caused to the readers by this change.
